# Supplementary material for: Functional ERAP1 Variants Distinctively Associate with Ankylosing Spondylitis Susceptibility under the Influence of HLA-B27 in Taiwanese
Source: Cells. 2022 Aug 5;11(15):2427. doi: 10.3390/cells11152427 (PMC9368314; doi:10.3390/cells11152427)
Supplement: Supplementary file 1 [file cells-11-02427-s001.zip › cells-1784262-supplementary.pdf]

Functional *ERAP1* variants distinctively associate with ankylosing spondylitis susceptibility under the influence of *HLA-B27* in Taiwanese

Chin-Man Wang*[1] MD, Ming-Kun Liu*[2] PhD, Yeong-Jian Jan Wu[2] MD, Jing-Chi Lin[2] MD, Jian-Wen Zheng[2] MS, Jianming Wu**[3] PhD, Ji-Yih Chen**[2] MD

Online Supplemental Methods

Supplemental Table S1. *ERAP1* SNVs and allelic variants (SNV haplotypes) in Taiwanese healthy controls and AS patients

Supplemental Table S2. Association of *ERAP1* SNVs with AS susceptibility in Taiwanese

Supplemental Table S3. Association of *ERAP1* SNVs with HLA-B27 positivity among AS patients

Supplemental Table S4. Distributions of *ERAP1* allelic variants in HLA-B27^{+} AS patients (AS B27^{+}) and HLA-B27^{+} healthy controls (Control B27^{+})

Supplemental Table S5. Distributions of HLA-B alleles in 62 HLA-B27^{-} AS patients

Supplemental Figure S1. Pair-wise LD patterns with D' (left side) and r2 (right side) measures of eleven ERAP1 SNPs for healthy controls (A) and AS cases plus normal (B).

Supplemental Figure S2. *ERAP1* allelic variants detected among different populations

Supplemental Figure S3. The original Western blots of Figures 1A, 2B and 3A

Supplemental Table S1. *ERAP1* SNVs and allelic variants (SNV haplotypes) in Taiwanese healthy controls and AS patients

SNV#	SNV ID	Codon Changes	Residue Change	Exon/ Intron	*ERAP1* allelic variant**					
					001	002	003	004	005	006
1	rs3734016G>A	GAG/AAG	E56K	Exon 2	G (E)	-	A (K)	-	-	-
2	rs26653G>C	CGT/CCT	R127P	Exon 2	G (R)	C (P)	C (P)	-	C (P)	-
3	rs26618A>G	ATA/ATG	I276M	Exon 5	A (I)	G (M)	-	-	-	-
4	rs2287987A>G	ATG/GTG	M349V	Exon 6	A (M)	-	-	-	G (V)	-
5	rs30187A>G	AAG/AGG	K528R	Exon 11	A (K)	G (R)	G (R)	-	G (R)	G (R)
6	rs10050860G>A	GAC/AAC	D575N	Exon 12	G (D)	-	-	-	A (N)	-
7	rs469783G>A*	GCG/GCA	A637A	Exon 13	G (A)	A (A)	-	-	-	-
8	rs17482078G>A	CGA/CAA	R725Q	Exon 15	G (R)	-	-	-	A (Q)	-
9	rs27044C>G	CAA/GAA	Q730E	Exon 15	C (Q)	G (E)	G (E)	-	G (E)	G (E)
10	rs27980A>C	A/C	-	3'-Intron	*A*	*C*	*C*	*C*	-	-
11	rs27037T>G	T/G	-	3'-Intron	*T*	*G*	*G*	*G*	*G*	*G*

*rs469783G>A is a silent coding SNV.

** Nucleotide substitutions (residue changes in parenthesis) are listed using the *ERAP1*-001 as the reference variant. Dashes "-" indicate nucleotides and residues are the same as *ERAP1*-001.

Supplemental Table S2. Association of *ERAP1* SNVs with AS susceptibility in Taiwanese

SNV	Risk Allele Frequency	Genotype Frequency			P_{Trend}*	P_{FDR}	Test model unadjusted				Test model adjusted for sex		
								P	P_{FDR}	OR (95% CI)	P	P_{FDR}	OR (95% CI)
rs3734016G>A	G	AA	AG	GG			Additive	0.1045	0.1437	1.16(0.97-1.40)	0.9404	0.9403	0.99(0.80-1.23)
case	1457(87.35%)	7(0.84%)	197(23.62%)	630(75.54%)	0.1055	0.1451	GG+AGvs AA	0.1018	0.1400	2.03(0.87-4.72)	0.3077	0.4231	1.71(0.61-4.82)
control	2438(85.66%)	24(1.69%)	360(25.30%)	1039(73.01%)			GG vs AG+AA	0.1873	0.2289	1.14(0.94-1.39)	0.7436	0.8180	0.96(0.76-1.21)
**rs26653G>C**	G	GG	CG	CC			Additive	0.0008	0.0017	1.24(1.09-1.40)	**0.0376**	**0.0689**	**1.17(1.01-1.35)**
case	1024(59.95%)	301(35.25%)	422(49.41%)	131(15.34%)	**0.0011**	**0.0024**	GG+CG vs CC	0.0085	0.0134	1.36(1.08-1.70)	0.1469	0.2308	1.22(0.93-1.59)
control	1564(54.92%)	421(29.56%)	722(50.70%)	281(19.73%)			GG vs CG+CC	0.0048	0.0089	1.30(1.08-1.55)	0.0593	0.1630	1.23(0.99-1.52)
rs26618A>G	A	GG	AG	AA			Additive	0.0742	0.1166	1.14(0.99-1.31)	0.0811	0.1275	1.16(0.98-1.36)
case	1311(76.67%)	45(5.26%)	309(36.14%)	501(58.60%)	0.0736	0.1157	AA+AG vs GG	0.2467	0.3015	1.24(0.86-1.79)	0.2503	0.3933	1.28(0.84-1.94)
control	2118(74.32%)	92(6.46%)	548(38.46%)	785(55.09%)			AA vs AG+GG	0.1020	0.1402	1.15(0.97-1.37)	0.1109	0.2034	1.18(0.96-1.44)
rs2287987A>G	A	GG	AG	AA			Additive	0.1982	0.2423	1.23(0.90-1.69)	0.2114	0.2907	1.26(0.88-1.81)
case	1615(96.36%)	0(0.00%)	61(7.28%)	777(92.72%)	0.2148	0.2625	AA+AG vs GG	0	0	NA	0.8426	0.8426	6557.58(0.00-32)
control	2705(95.58%)	1(0.07%)	123(8.69%)	1291(91.24%)			AA vs AG+GG	0.2156	0.2371	1.22(0.89-1.68)	0.2448	0.3366	1.24(0.86-1.79)
**rs30187A>G**	A	GG	AG	AA			Additive	9.28E-05	0.0003	1.27(1.13-1.44)	**0.0125**	**0.0343**	**1.20(1.04-1.38)**
case	995(57.92%)	149(17.35%)	425(49.48%)	285(33.18%)	**<0.0001**	**<0.0001**	AA+AG vs GG	0.0017	0.0038	1.41(1.14-1.75)	0.1097	0.2011	1.23(0.95-1.58)
control	1493(51.98%)	328(22.84%)	723(50.35%)	385(26.81%)			AA vs AG+GG	0.0012	0.0033	1.36(1.13-1.63)	0.0158	0.0579	1.31(1.05-1.62)
rs10050860G>A	G	GG	AG	AA			Additive	0.2642	0.2907	1.19(0.88-1.62)	0.2858	0.3494	1.21(0.85-1.72)
case	1639(96.19%)	787(92.37%)	65(7.63%)	0(0.00%)	0.2953	0.3248	GG+AG vs AA	0	0	Inf(Inf- Inf)	0.9695	0.9695	Inf(0.00- Inf)
control	2716(95.50%)	1297(91.21%)	122(8.58%)	3(0.21%)			GG vs AG+AA	0.3331	0.3664	1.17(0.85-1.60)	0.3853	0.4710	1.17(0.82-1.68)
**rs469783G>A**	G	GG	AG	AA			Additive	3.73E-07	4.10E-06	1.37(1.21-1.54)	**0.0005**	**0.0029**	**1.28(1.11-1.48)**
case	991(58.02%)	285(33.37%)	421(49.30%)	148(17.33%)	**<0.0001**	**<0.0001**	GG+AG vs AA	1.57E-06	8.63E-06	1.69(1.36-2.09)	0.0027	0.0295	1.46(1.14-1.87)
control	1428(50.11%)	375(26.32%)	678(47.58%)	372(26.11%)			GG vs AG+AA	0.0003	0.0009	1.40(1.17-1.69)	0.0071	0.0393	1.35(1.08-1.68)
rs17482078G>A	G	GG	AG	AA			Additive	0.3732	0.3732	1.15(0.85-1.55)	0.4335	0.4769	1.15(0.81-1.63)

case	1643(96.08%)	789(92.28%)	65(7.60%)	1(0.12%)	0.395	0.395	GG+AG vs AA	0.6087	0.6087	1.81(0.19-17.40)	0.5243	0.6408	2.17(0.20-23.52)
control	2715(95.53%)	1297(91.27%)	121(8.52%)	3(0.21%)			GG vs AG+AA	0.4006	0.4006	1.14(0.84-1.56)	0.4803	0.5283	1.14(0.79-1.63)
**rs27044C>G**	C	GG	CG	CC			Additive	6.68E-06	2.45E-05	1.32(1.17-1.50)	**0.0027**	**0.0010**	**1.25(1.08-1.44)**
case	979(56.92%)	154(17.91%)	433(50.35%)	273(31.74%)	**<0.0001**	**<0.0001**	CC+CG vs GG	0.0003	0.0009	1.48(1.20-1.83)	0.0269	0.0986	1.32(1.03-1.69)
control	1439(50.10%)	350(24.37%)	733(51.04%)	353(24.58%)			CC vs CG+GG	0.0002	0.0007	1.43(1.18-1.72)	0.0084	0.0461	1.35(1.08-1.68)
**rs27980A>C**	A	AA	AC	CC			Additive	0.0063	0.0115	1.18(1.05-1.34)	**0.0203**	**0.0446**	**1.17(1.03-1.34)**
case	988(57.38%)	277(32.17%)	434(50.41%)	150(17.42%)	**0.0053**	**0.0097**	AA+AC vs CC	0.0070	0.0128	1.35(1.08-1.67)	0.0392	0.0863	1.29(1.01-1.63)
control	1531(53.23%)	411(28.58%)	709(49.30%)	318(22.11%)			AA vs AC+CC	0.0690	0.1084	1.19(0.99-1.42)	0.0805	0.1771	1.20(0.98-1.47)
**rs27037T>G**	T	GG	GT	TT			Additive	6.08E-06	2.45E-05	1.33(1.17-1.50)	**0.0004**	**0.0029**	**1.30(1.12-1.50)**
case	821(48.12%)	220(25.79%)	445(52.17%)	188(22.04%)	<0.0001	<0.0001	TT+GTvs GG	2.11E-05	0.0001	1.50(1.25-1.82)	0.0005	0.0056	1.47(1.18-1.83)
control	1186(41.30%)	493(34.33%)	700(48.75%)	243(16.92%)			TT vs GT+GG	0.0025	0.0055	1.39(1.12-1.72)	0.0253	0.0696	1.33(1.04-1.71)

*P_{Trend} indicates the *P*-value from the Cochran–Armitage trend test with 10,000 permutations. Six SNVs (rs26653, rs30187, rs469783, rs27044, rs27980, and rs27037) highlighted in bold were significantly associated with AS.

Supplemental Table S3. Association of *ERAP1* SNVs with HLA-B27 positivity among AS patients

SNV	Risk Allele Frequency	Genotype Frequency			P_{Trend}*	P_{FDR}	Test model unadjusted				Test model adjusted for sex		
								P	P_{FDR}	OR (95% CI)	P	P_{FDR}	OR (95% CI)
rs3734016G>A	A	AA	AG	GG			Additive	0.6746	0.6746	1.14(0.63-2.06)	0.6435	0.8848	1.15(0.63-2.12)
HLA-B27+	197(12.74%)	6(0.78%)	185(23.93%)	582(75.29%)	0.7751	0.7751	AA+AG vs GG	0.5528	0.5528	1.21(0.64-2.28)	0.4981	0.6087	1.25(0.66-2.37)
HLA-B27-	14(11.48%)	1(1.64%)	12(19.67%)	48(78.69%)			AA vs AG+GG	0.4871	0.5781	0.47(0.06-3.96)	0.3051	0.3729	0.32(0.04-2.80)
**rs26653G>C**	G	GG	CG	CC			Additive	0.0032	0.0089	1.75(1.21-2.54)	**0.0042**	**0.0115**	**1.73(1.19-2.52)**
HLA-B27+	964(60.94%)	283(35.78%)	398(50.32%)	110(13.91%)	**0.0028**	**0.0083**	GG+CG vs CC	7.94E-05	0.0004	3.10(1.77-5.43)	0.0001	0.0007	3.03(1.72-5.36)
HLA-B27-	60(47.62%)	18(28.57%)	24(38.10%)	21(33.33%)			GG vs CG+CC	0.2510	0.4174	1.39(0.79-2.45)	0.2550	0.3729	1.39(0.79-2.46)
**rs26618A>G**	A	GG	AG	AA			Additive	0.0004	0.0039	2.03(1.38-3.01)	**0.0002**	**0.0021**	**2.12(1.43-3.14)**
HLA-B27+	1231(77.71%)	38(4.80%)	277(34.97%)	477(60.23%)	**0.0004**	**0.0044**	AA+AG vs GG	0.0363	0.0800	2.48(1.06-5.81)	0.0331	0.0728	2.55(1.08-6.04)
HLA-B27-	80(63.49%)	7(11.11%)	32(50.79%)	24(38.10%)			AA vs AG+GG	0.0008	0.0021	2.46(1.45-4.17)	0.0004	0.0012	2.62(1.53-4.48)
rs2287987A>	A	GG	AG	AA			Additive	0.4775	0.6566	1.38(0.57-3.34)	0.7533	0.9207	1.16(0.47-2.88)
HLA-B27+	1495(96.45%)	0(0.00%)	55(7.10%)	720(92.90%)	0.6073	0.6954	AA+AG vs GG	8.47E-82	9.32E-81	3.51(3.08-3.99)	1.51E-24	1.66E-23	6.60(4.60-9.48)
HLA-B27-	120(95.24%)	0(0.00%)	6(9.52%)	57(90.48%)			AA vs AG+GG	0.4775	0.5306	1.38(0.57-3.34)	0.7533	0.8287	1.16(0.47-2.88)
**rs30187A>G**	A	GG	AG	AA			Additive	0.0027	0.0089	1.74(1.21-2.51)	**0.0027**	**0.0099**	**1.74(1.21-2.51)**
HLA-B27+	935(58.95%)	126(15.89%)	399(50.32%)	268(33.80%)	**0.003**	**0.0083**	AA+AG vs GG	0.0002	0.0009	2.83(1.65-4.86)	0.0003	0.0015	2.77(1.60-4.80)
HLA-B27-	60(45.45%)	23(34.85%)	26(39.39%)	17(25.76%)			AA vs AG+GG	0.1849	0.2642	1.47(0.83-2.60)	0.1487	0.2337	1.53(0.86-2.73)
rs10050860G>A	G	GG	AG	AA			Additive	0.5571	0.6721	1.30(0.54-3.15)	0.8754	0.9482	1.08(0.43-2.67)
HLA-B27+	1519(96.26%)	730(92.52%)	59(7.48%)	0(0.00%)	0.6124	0.6954	GG+AG vs AA	NaN	NaN	1.00(1.00-1.00)	9.88E-25	1.09E-23	0.02(0.01-0.05)
HLA-B27-	120(95.24%)	57(90.48%)	6(9.52%)	0(0.00%)			GG vs AG+AA	0.5571	0.5781	1.30(0.54-3.15)	0.8754	0.9083	1.08(0.43-2.67)
**rs469783G>A**	G	GG	AG	AA			Additive	0.0048	0.0106	1.70(1.18-2.46)	**0.0057**	**0.0126**	**1.69(1.16-2.44)**
HLA-B27+	933(58.98%)	268(33.88%)	397(50.19%)	126(15.93%)	**0.0045**	**0.0099**	GG+AG vs AA	0.0002	0.0007	2.83(1.63-4.92)	0.0004	0.0012	2.77(1.59-4.85)
HLA-B27-	58(46.03%)	17(26.98%)	24(38.10%)	22(34.92%)			GG vs AG+AA	0.2656	0.4174	1.39(0.78-2.47)	0.2530	0.3729	1.40(0.78-2.51)

rs17482078G>A	G	GG	AG	AA			Additive	0.6110	0.6721	1.25(0.53-2.97)	0.9482	0.9482	1.03(0.42-2.51)
HLA-B27+	1523(96.15%)	732(92.42%)	59(7.45%)	1(0.13%)	0.6322	0.6954	GG+AG vs AA	1.73E-06	1.73E-05	0.00(0.00-0.00)	0.8912	0.8912	0.00(0.00-12)
HLA-B27-	120(95.24%)	57(90.48%)	6(9.52%)	0(0.00%)			GG vs AG+AA	0.5781	0.5781	1.28(0.53-3.10)	0.9083	0.9083	1.05(0.43-2.62)
**rs27044C>G**	C	GG	CG	CC			Additive	0.0017	0.0089	1.80(1.25-2.59)	**0.0017**	**0.0094**	**1.80(1.25-2.60)**
HLA-B27+	921(58.00%)	131(16.50%)	405(51.01%)	258(32.49%)	**0.0014**	**0.0077**	CC+CG vs GG	0.0003	0.0011	2.71(1.58-4.64)	0.0004	0.0016	2.67(1.54-4.62)
HLA-B27-	58(43.94%)	23(34.85%)	28(42.42%)	15(22.73%)			CC vs CG+GG	0.1044	0.1740	1.64(0.90-2.97)	0.0834	0.1529	1.70(0.93-3.11)
rs27980A>C	A	AA	AC	CC			Additive	0.0714	0.1121	1.40(0.97-2.00)	0.0576	0.0905	1.42(0.99-2.04)
HLA-B27+	922(57.99%)	259(32.58%)	404(50.82%)	132(16.60%)	0.083	0.1304	AA+AC vs CC	0.0303	0.0607	1.88(1.06-3.34)	0.0341	0.0750	1.87(1.05-3.35)
HLA-B27-	66(50.00%)	18(27.27%)	30(45.45%)	18(27.27%)			AA vs AC+CC	0.3763	0.5174	1.29(0.73-2.26)	0.2817	0.3729	1.37(0.77-2.42)
**rs27037T>G**	T	GG	GT	TT			Additive	0.0198	0.0363	1.57(1.07-2.28)	**0.0147**	**0.0270**	**1.60(1.10-2.34)**
HLA-B27+	771(48.92%)	193(24.49%)	419(53.17%)	176(22.34%)	**0.0201**	**0.0368**	TT+GTvs GG	0.0031	0.0085	2.19(1.30-3.68)	0.0029	0.0079	2.23(1.32-3.77)
HLA-B27-	50(38.46%)	27(41.54%)	26(40.00%)	12(18.46%)			TT vs GT+GG	0.4699	0.5306	1.27(0.66-2.43)	0.3659	0.5032	1.35(0.70-2.61)

* P_{Trend} indicates the *P*-value from the Cochran–Armitage trend test with 10,000 permutations. Six SNVs (rs26653, rs26618, rs30187, rs469783, rs27044, and rs27037) highlighted in bold were significantly associated with HLA-B27 positivity among AS patients.

Supplemental Table S4. Distributions of *ERAP1* allelic variants in HLA-B27$^+$ AS patients (AS B27$^+$) and HLA-B27$^+$ healthy controls (Control B27$^+$)

ERAP1* allelic variant	**Estimated Frequency**			**Permutation****	**Logistic regression**		**Logistic regression adjusted for sex**	
	**AS B27$^+$** (2N=1602)	**Control B27$^+$** (2N=136)	**All** (2N=1734)	**P value**	**P value**	**OR (95% CI)**	**P value**	**OR (95% CI)**
001	47.68%	40.21%	47.10%	0.0798	0.0833	1.39(0.96-2.01)	0.1163	1.36(0.93-1.99)
002	20.89%	22.34%	21.00%	0.692	0.6848	0.92(0.60-1.40)	0.7133	0.92(0.60-1.43)
003	12.26%	14.37%	12.43%	0.4476	0.4472	0.81(0.47-1.39)	0.2324	0.71(0.40-1.25)
004	8.07%	6.78%	7.97%	0.5983	0.5933	1.21(0.60-2.43)	0.5304	1.26(0.62-2.56)
005	4.03%	3.52%	3.99%	0.7602	0.7581	1.17(0.43-3.15)	0.7075	1.22(0.44-3.38)
006	2.11%	2.93%	2.17%	0.4853	0.5211	0.70(0.24-2.07)	0.6027	0.74(0.24-2.29)

*Eleven SNVs including rs3734016, rs26653, rs26618, rs2287987, rs30187, rs10050860, rs469783, rs17482078, rs27044, rs27980, and rs27037 were used to determine *ERAP1* variants as shown in the Supplemental Table 1.

***P*-values for *ERAP1* variants were generated using the expectation-maximization (EM) algorithm with 10,000 permutations.

Supplemental Table S5. Distributions of *HLA-B* alleles in HLA-B27^{-} AS patients

HLA-B allele	Phenotype frequency (N=62)	Allele frequency (2N=124)
HLA-B1301	12 (19.35%)	13 (10.48%)
HLA-B1302	1 (1.61%)	1 (0.81%)
HLA-B1501	5 (8.06%)	5 (4.03%)
HLA-B1502	6 (9.68%)	6 (4.84%)
HLA-B1512	1 (1.61%)	1 (0.81%)
HLA-B1518	1 (1.61%)	1 (0.81%)
HLA-B3501	4 (6.45%)	4 (3.23%)
HLA-B3503	1 (1.61%)	1 (0.81%)
HLA-B3801	1 (1.61%)	1 (0.81%)
HLA-B3802	6 (9.68%)	6 (4.84%)
HLA-B3842	1 (1.61%)	1 (0.81%)
HLA-B3901	6 (9.68%)	6 (4.84%)
HLA-B3909	1 (1.61%)	1 (0.81%)
HLA-B4001	23 (37.10%)	23 (18.55)
HLA-B4401	2 (3.23%)	2 (1.61%)
HLA-B4402	1 (1.61%)	1 (0.81%)
HLA-B4403	1 (1.61%)	1 (0.81%)
HLA-B4601	17 (27.42%)	18 (14.52%)
HLA-B4801	2 (3.23%)	2 (1.61%)
HLA-B5101	4 (6.45%)	4 (3.22%)
HLA-B5102	4 (6.45%)	4 (3.22%)
HLA-B5401	4 (6.45%)	4 (3.23%)
HLA-B5501	1 (1.61%)	1 (0.81%)
HLA-B5502	3 (4.84%)	3 (2.42%)
HLA-B5504	1 (1.61%)	1 (0.81%)
HLA-B5601	3 (4.84%)	3 (2.42%)
HLA-B5801	9 (14.52%)	9 (7.26%)
HLA-B6701	1 (1.61%)	1 (0.81%)

Supplemental Figure S1. Linkage disequilibrium (LD) analysis of *ERAP1* SNVs. Haploview 4.2 was used to calculate LD between marker loci. (A). Pair-wise LD patterns show D’ (left panel) and r^2 (right panel) values of eleven *ERAP1* SNVs in Taiwanese healthy controls. (B) Pair-wise LD patterns show D’ (left panel) and r^2 (right panel) values of eleven ERAP1 SNVs in the combined Taiwanese population of AS cases and healthy controls.

A.

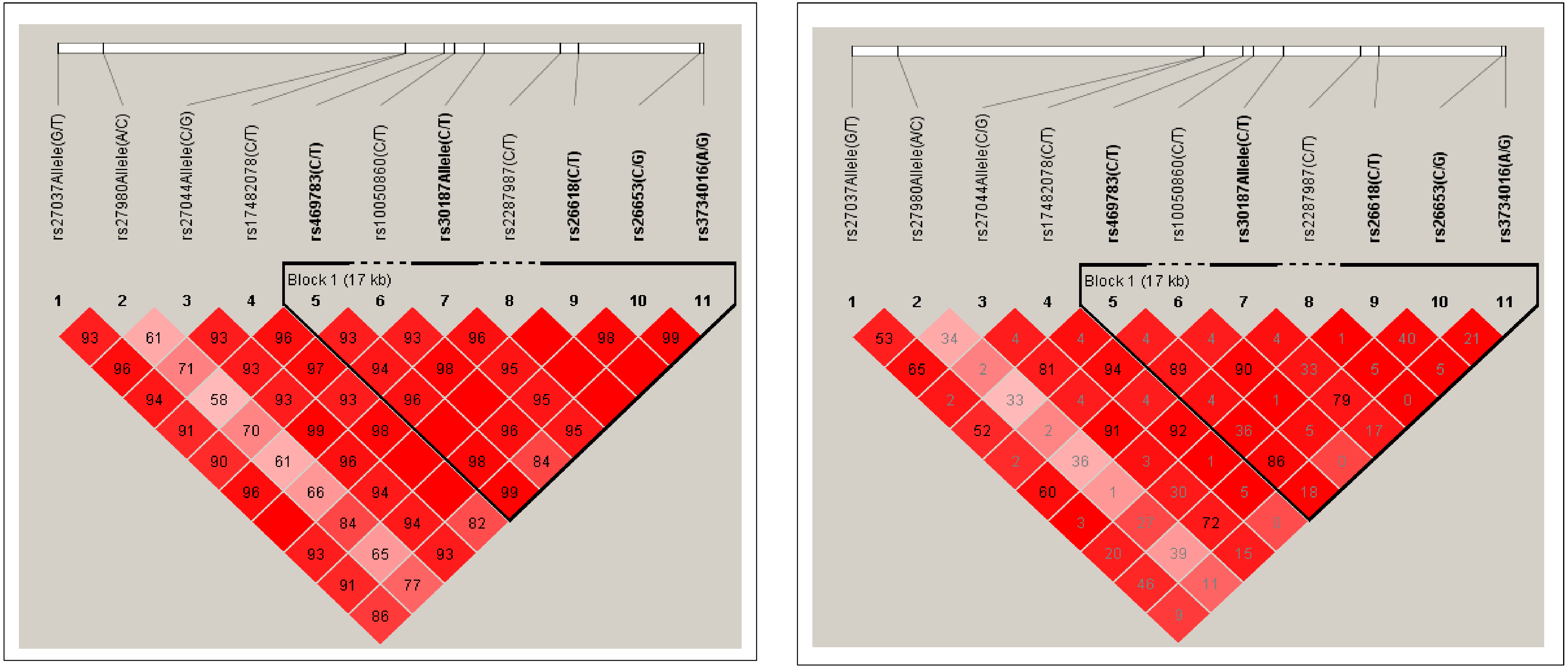

B.

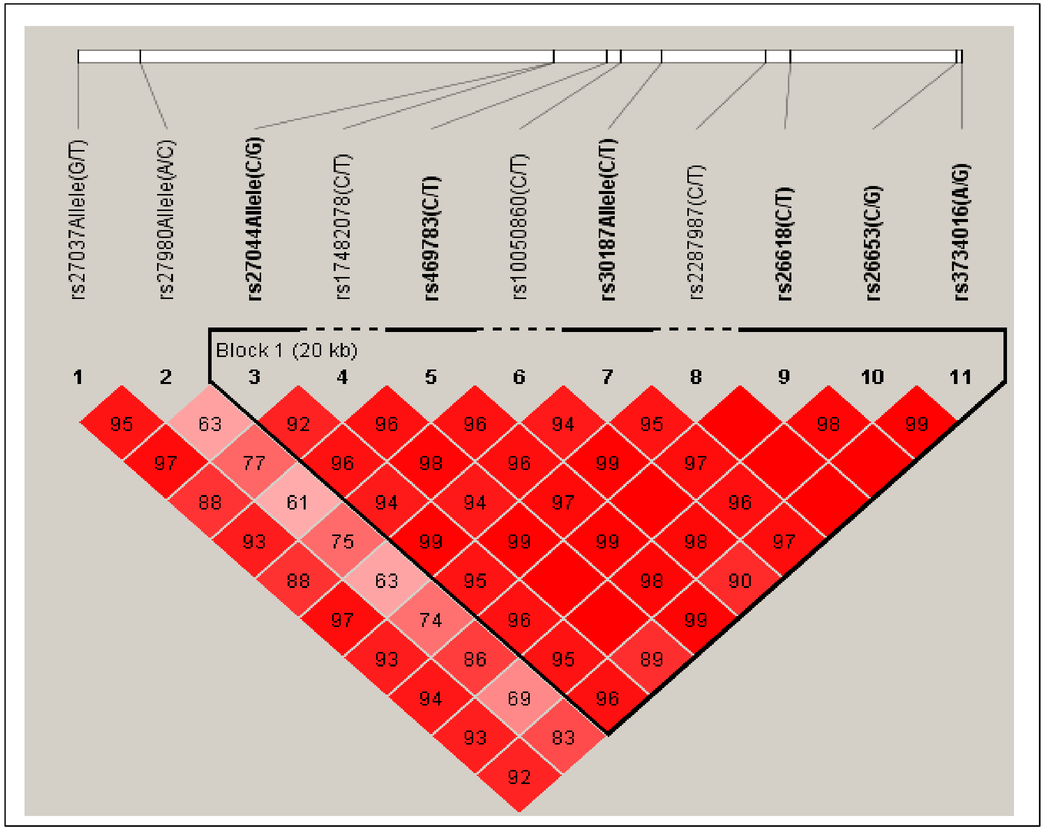
rs27037Allele(G/T)
rs27980Allele(A/C)
rs27044Allele(C/G)
rs17482078(C/T)
rs469783(C/T)
rs10050860(C/T)
rs30187Allele(C/T)
rs2287987(C/T)
rs26618(C/T)
rs26653(C/G)
rs3734016(A/G)
Block 1 (20 kb)

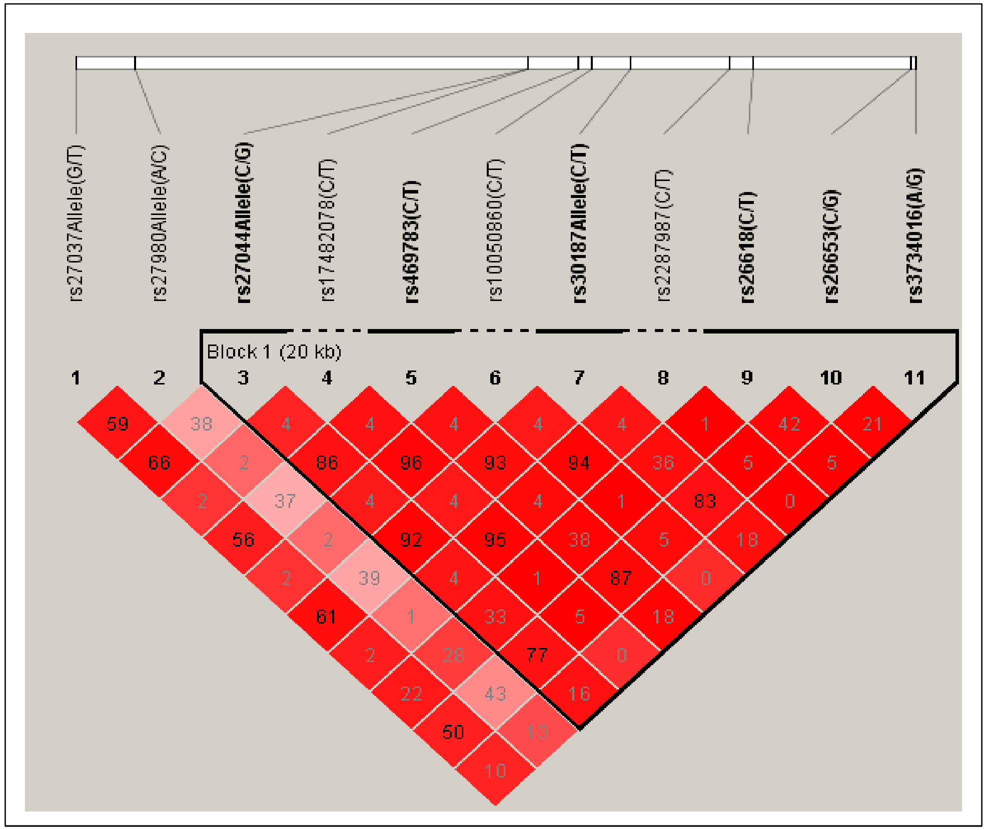
rs27037Allele(G/T)
rs27980Allele(A/C)
rs27044Allele(C/G)
rs17482078(C/T)
rs469783(C/T)
rs10050860(C/T)
rs30187Allele(C/T)
rs2287987(C/T)
rs26618(C/T)
rs26653(C/G)
rs3734016(A/G)
Block 1 (20 kb)

Supplemental Figure S2. *ERAP1* SNVs in human populations.

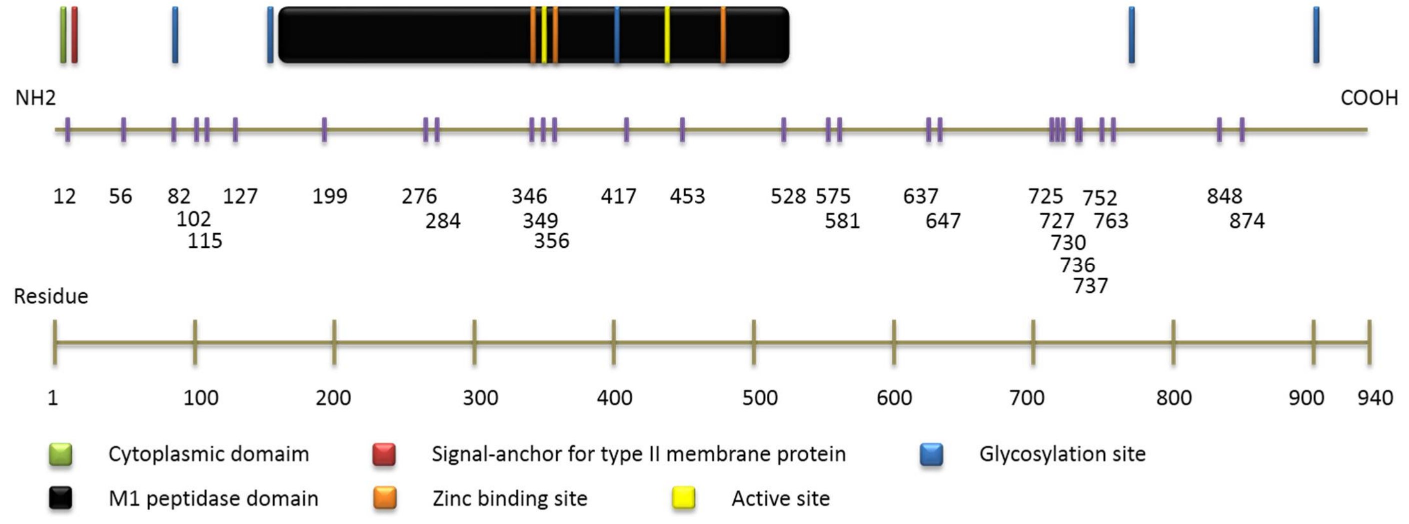

Amino acid at indicated position																													
12	56	82	102	115	127	199	276	284	346	349	356	417	453	528	575	581	637	647	725	727	730	736	737	752	763	848	874	Ethnicity	Refs
	○				○		○			●				●	●		○		●		●							Asian	Chen et al
					○		○	○	○	●	○	○	○	●	●		○	○	●		●	○			○	○		European	Harvey et al., 2009
					○		○		○	●				●	●				●		●							Cell lines	García-Medel et al., 2012
					○					●			○	●	●				●		●							European	Cinar et al., 2013
		○	○	○	○	○				●				●	●	○			●	○	●		○	○			○	European	Reeves et al., 2014
○	○				○		○		○	●				●	●				●		●							1000 Genomes Project super-populations	Ombrello et al., 2015
										●				●	●				●		●							European	Roberts et al., 2017

Upper panel: ERAP1 domains (or sites) are indicated with different colors. Amino acid positions of SNVs detected in human populations are shown as vertical purple bars.

Lower panel: Summary of ERAP1 SNVs in different human populations. Black circle indicated the SNVs used for haplotype analyses in most genetic studies.

Figure 1A

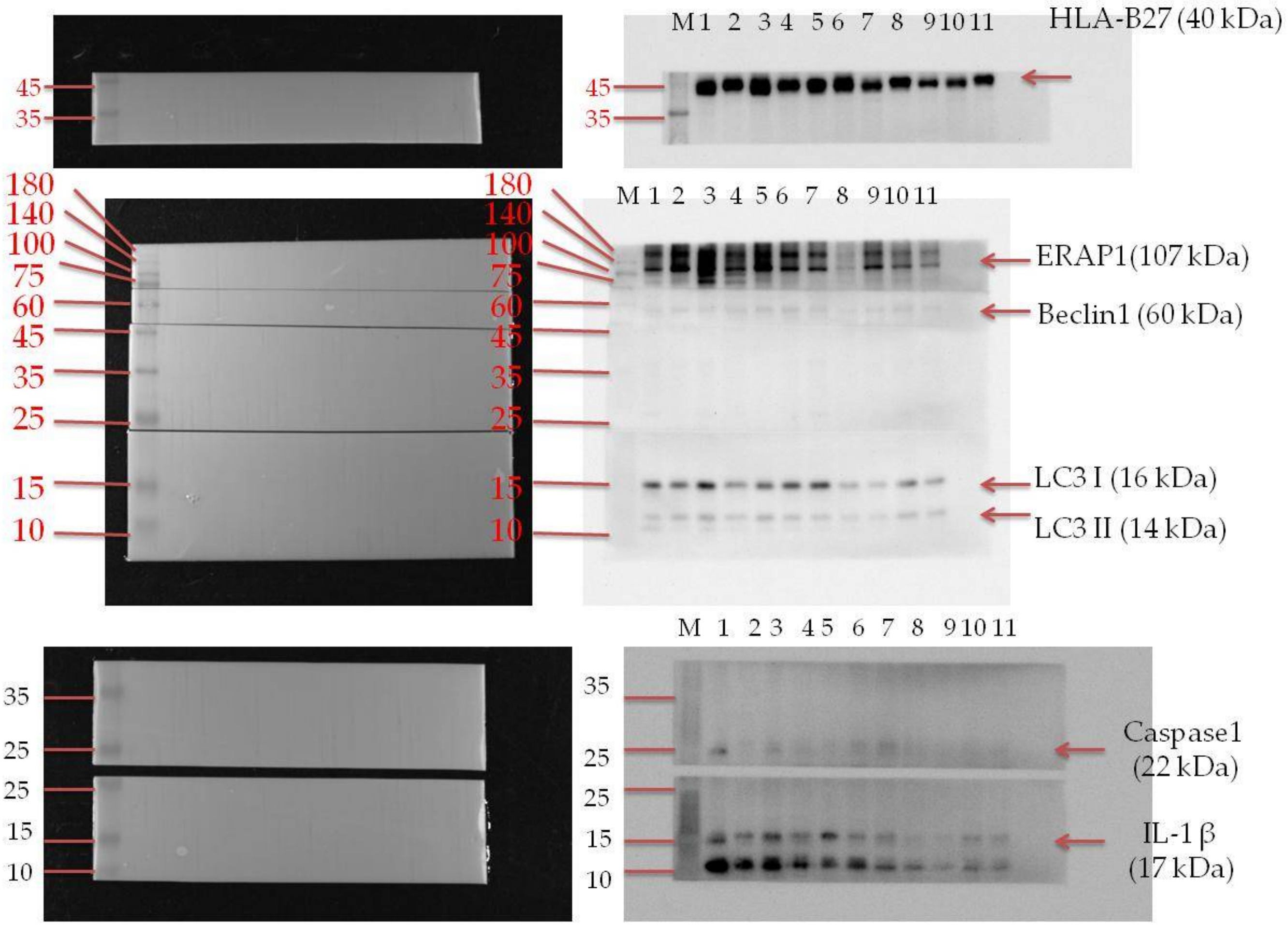
HLA-B27 (40 kDa)
M 1 2 3 4 5 6 7 8 9 10 11
45
35
180
140
100
75
60
45
35
25
15
10
ERAP1(107 kDa)
Beclin1 (60 kDa)
LC3 I (16 kDa)
LC3 II (14 kDa)
Caspase1 (22 kDa)
IL-1 β (17 kDa)

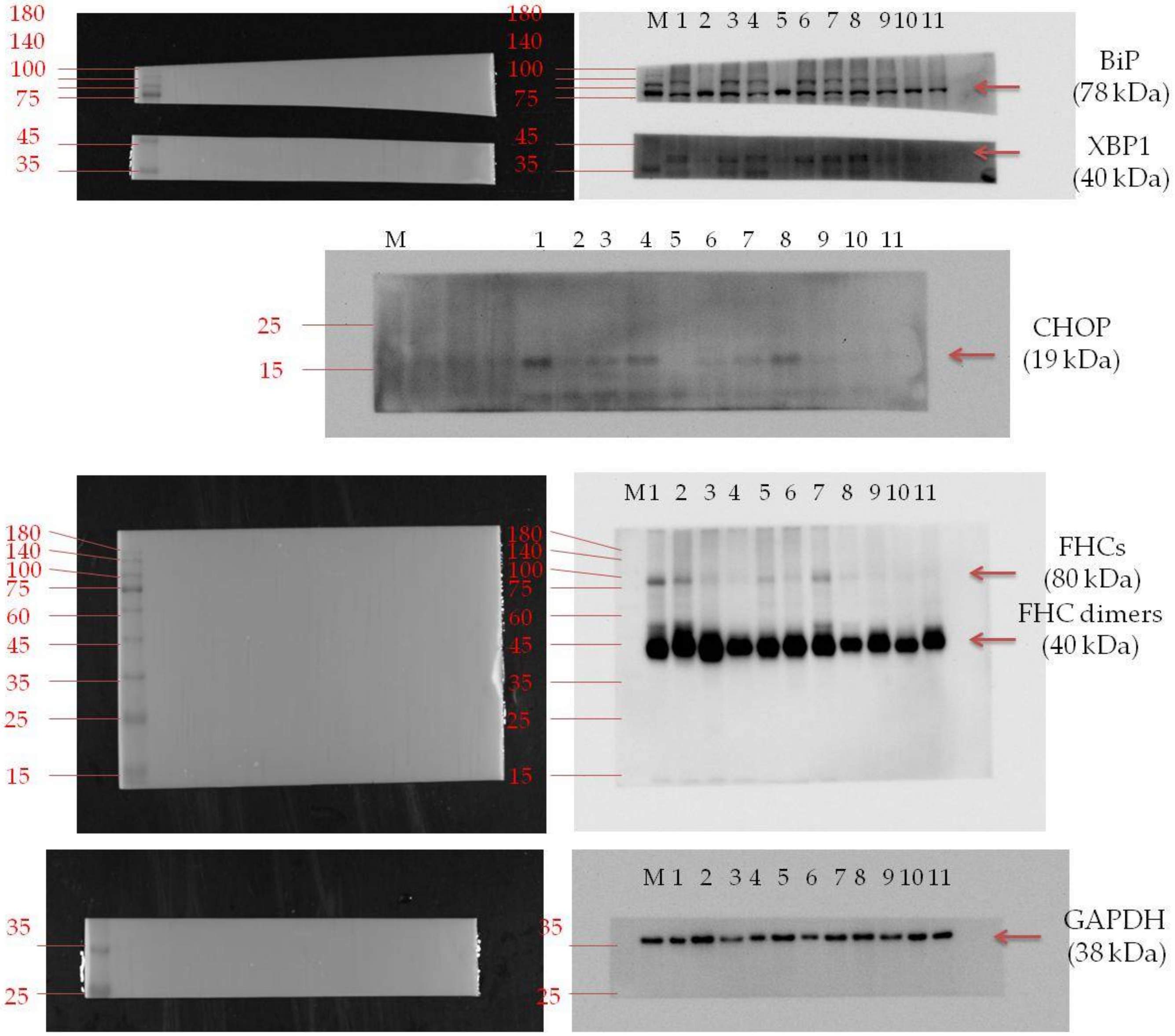
M 1 2 3 4 5 6 7 8 9 10 11
180
140
100
75
45
35
BiP
(78 kDa)
XBP1
(40 kDa)
M 1 2 3 4 5 6 7 8 9 10 11
25
15
CHOP
(19 kDa)
M 1 2 3 4 5 6 7 8 9 10 11
180
140
100
75
60
45
35
25
15
FHCs
(80 kDa)
FHC dimers
(40 kDa)
M 1 2 3 4 5 6 7 8 9 10 11
35
25
GAPDH
(38 kDa)

Figure 2B

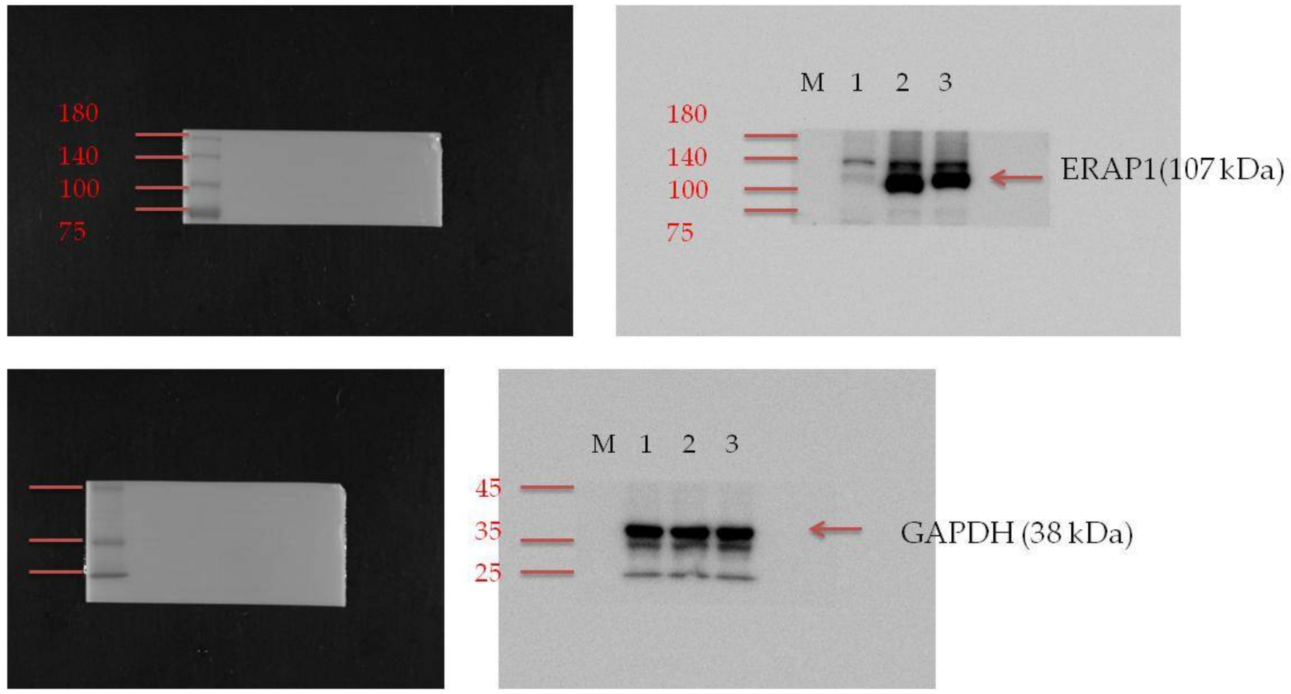
M 1 2 3
180
140
100
75
ERAP1(107 kDa)
M 1 2 3
45
35
25
GAPDH (38 kDa)

M: marker, 1: Vector, 2: ERAP1-001, 3: ERAP1-002

Figure 3A

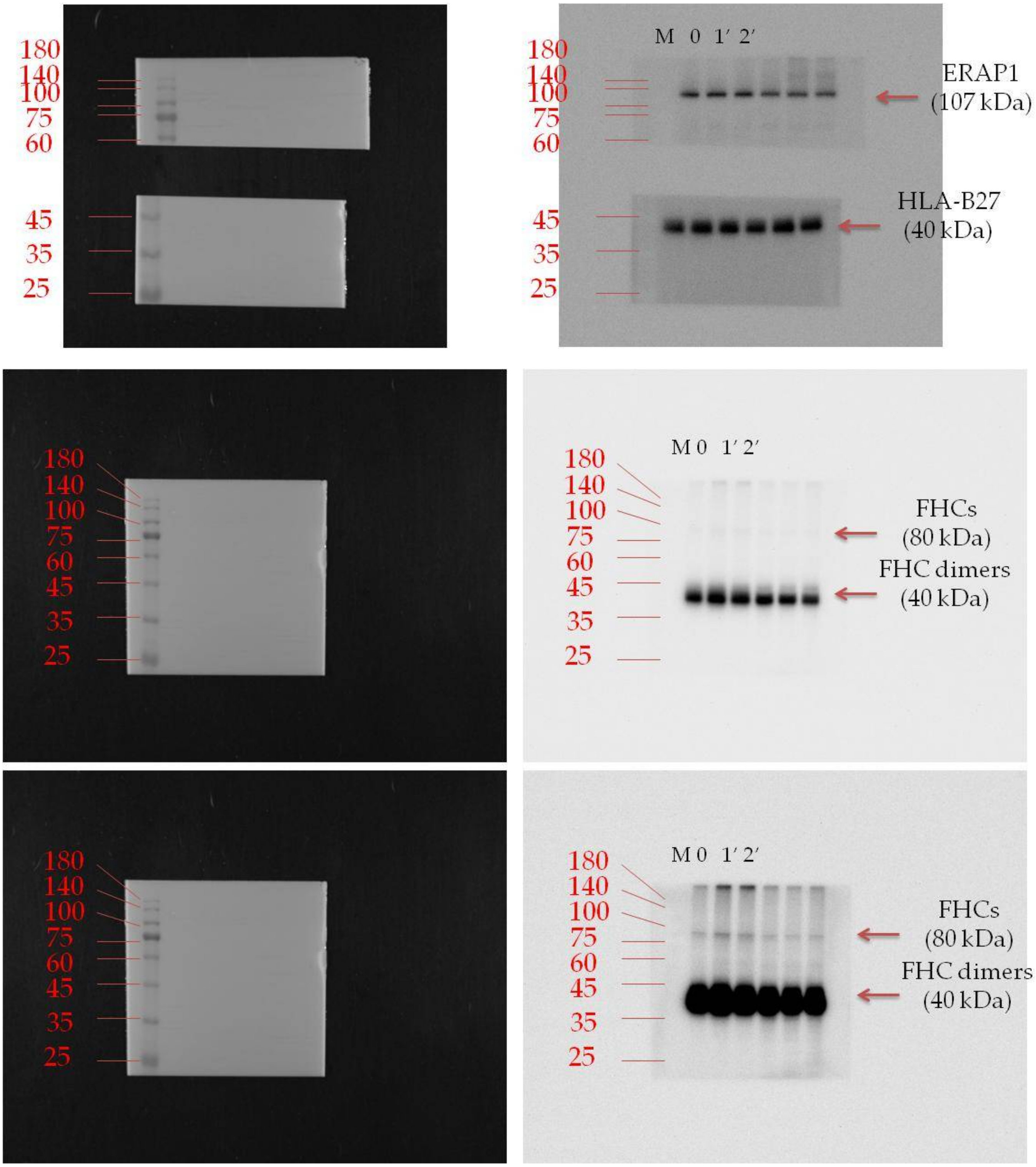

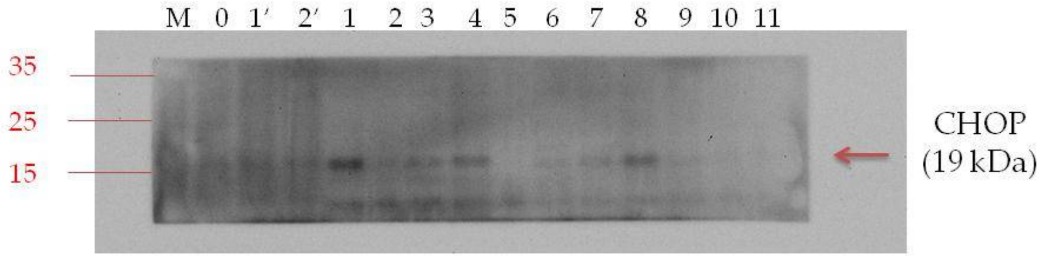
M 0 1′ 2′ 1 2 3 4 5 6 7 8 9 10 11
35
25
15
CHOP
(19 kDa)

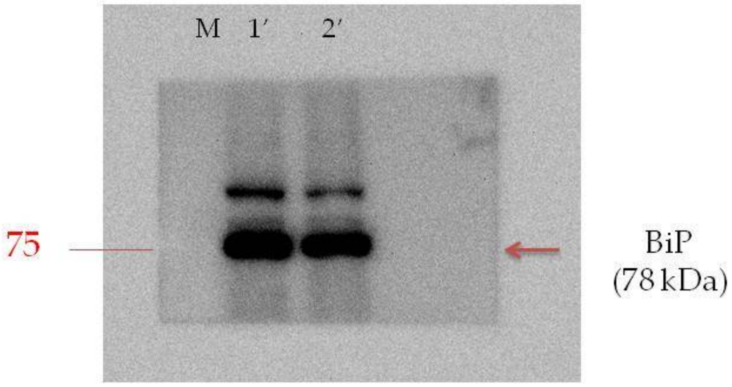
M 1′ 2′
75
BiP
(78 kDa)

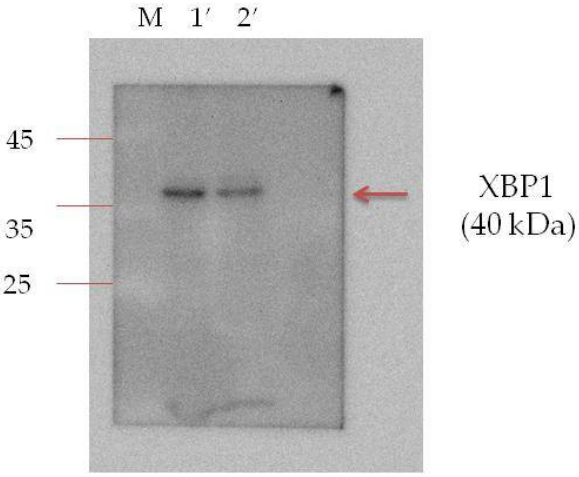
M 1′ 2′
45
35
25
XBP1
(40 kDa)

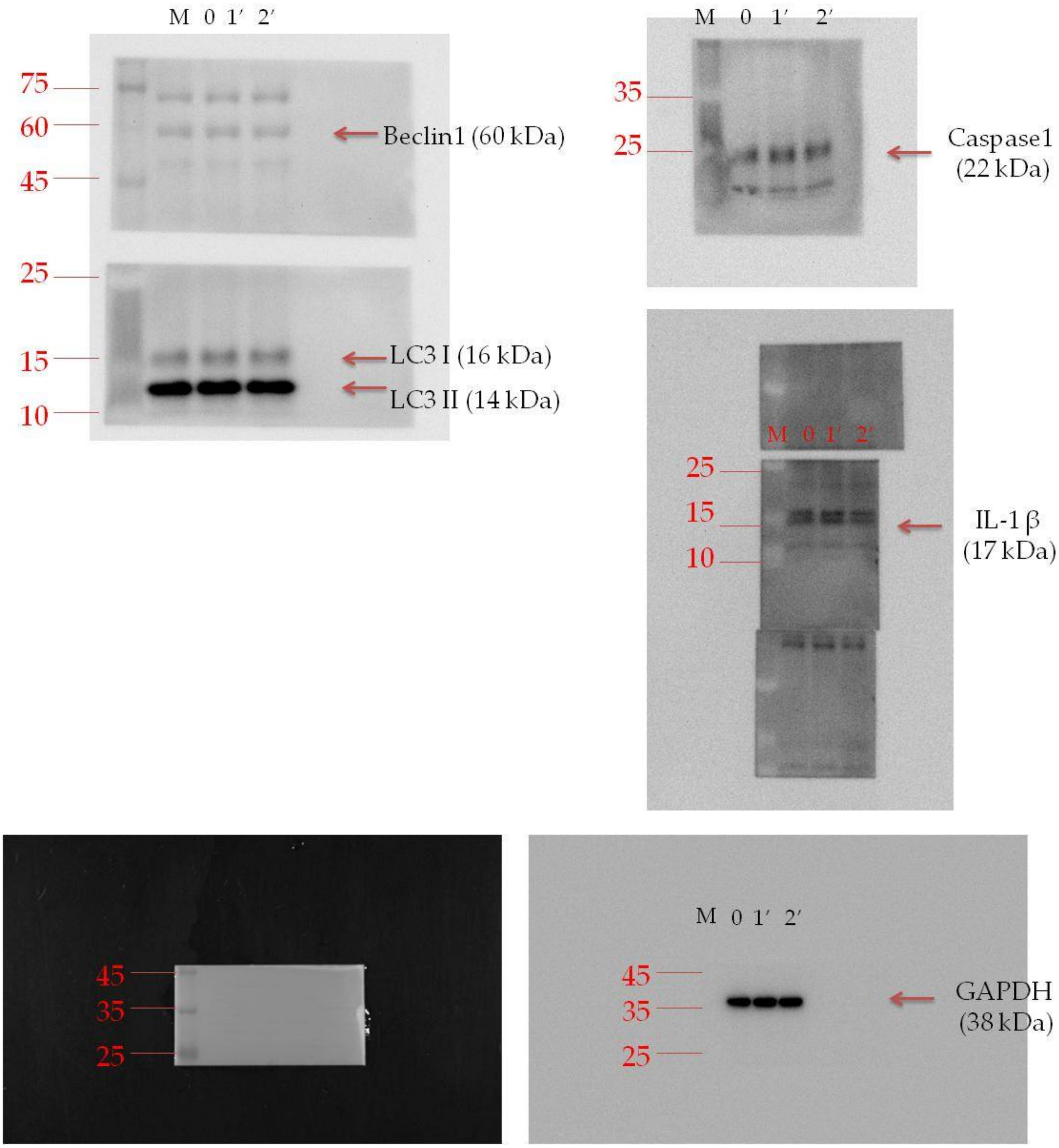

M: marker, 0: Vector, 1′: ERAP1-001, 2′: ERAP1-002
